# *Borrelia*, *Leishmania*, and *Babesia*: An Emerging Triad of Vector-Borne Co-Infections?

**DOI:** 10.3390/pathogens14010036

**Published:** 2025-01-06

**Authors:** Elianne Piloto-Sardiñas, Ana Laura Cano-Argüelles, Alejandro Cabezas-Cruz

**Affiliations:** 1 Direction of Animal Health, National Center for Animal and Plant Health, Carretera de Tapaste y Autopista Nacional, Apartado Postal 10, San José de las Lajas 32700, Cuba; 2 ANSES, INRAE, Ecole Nationale Vétérinaire d’Alfort, UMR BIPAR, Laboratoire de Santé Animale, F-94700 Maisons-Alfort, France; 3 Parasitology Laboratory, Institute of Natural Resources and Agrobiology of Salamanca (IRNASA, CSIC), Cordel de Merinas, 40-52, 37008 Salamanca, Spain; ana.cano@irnasa.csic.es

Canine leishmaniosis (CanL), caused by the protozoan *Leishmania infantum* and transmitted primarily by phlebotomine sand flies, poses significant challenges for zoonotic disease management [1], with dogs serving as reservoirs, facilitating transmission to humans [2]. Host exposure to sand fly vectors, as well as ticks carrying other pathogens, increases the risk of co-infection with *Leishmania*, *Borrelia*, and *Babesia* [3]. These co-infections may exacerbate CanL progression due to synergistic interactions between pathogens that manipulate host immune responses [3].

The dynamics of zoonotic diseases are increasingly influenced by the overlapping habitats of vectors such as sand flies and ticks [4,5,6]. Climate change and habitat alterations are driving these vectors into new territories [7,8], creating conditions in which ticks (carrying *Borrelia* and *Babesia*) and sand flies (carrying *Leishmania*) can coexist [9,10,11,12,13,14,15]. This overlap enhances the likelihood of hosts, particularly dogs, becoming co-infected with multiple pathogens [9,10,11,12,13,14,15,16]. Pathogen interactions during co-infections—synergistic, antagonistic, or neutral—affect virulence, pathogenicity, and colonization success [17,18], affecting disease progression, and ultimately the risk of human infection [11,19].

A recent study by Pessôa-Pereira et al. [4] highlights how *Borrelia burgdorferi*, the agent of Lyme disease, exacerbates *Leishmania* infection in co-infected dogs. This study underscores the broader implications of co-infections involving vector-borne pathogens with overlapping hosts and/or vectors. *Babesia*, a protozoan parasite transmitted by ticks, causes babesiosis [20] and has been reported in co-infections with *Leishmania* in dogs [3,9,21]. Similarly, in humans, co-infection with *Babesia* and *Borrelia* worsens the clinical course of both Lyme disease and babesiosis [22]. Given these findings, it raises the possibility that co-infection with *Borrelia* might similarly affect dogs with *Leishmania*, potentially enhancing CanL progression. This hypothesis is supported by the study of Pessôa-Pereira et al. [4], which showed that *Borrelia* co-infection increases *Leishmania* survival in macrophages by altering immune responses. Combined immune modulation by *Borrelia*, *Leishmania*, and *Babesia*—each employing different mechanisms to evade or manipulate host defenses [3]—could amplify disease severity and complicate treatment.

Despite their structural differences and distinct pathogenesis mechanisms, bacteria and protozoa can establish ecological interactions within biological systems [23,24]. In terrestrial (e.g., soil) or aquatic ecosystems, bacterial communities can interfere with protozoan colonization or predation, often fostering antagonistic relationships [23,24,25]. These antagonisms arise through intracellular mechanisms (e.g., survival and replication within protozoan cells) or extracellular adaptations (e.g., altered cell morphology, increased motility, or biofilm formation) [26], as well as the production of bacteriocins with antiprotozoal activity [27]. However, these interactions differ significantly when subjected to the biology, physiology, and immune pressures of hosts and vectors.

During co-infections in hosts or vectors, pathogens may interact at various stages of their life cycles, targeting the same cells or eliciting overlapping immune responses. For instance, shared immune cell tropism between vector-borne bacteria and protozoa or the modulation of immune functions, such as macrophage activity, can allow bacterial infections to enhance protozoal infections [11]. This overlap enables *Borrelia*, *Leishmania*, and *Babesia* to exploit host immune systems, exacerbating disease severity. For instance, *Borrelia* promotes a skewed Th17 immune response that is inflammatory but insufficient for clearance [28], *Leishmania* inhibits macrophage reactive oxygen species (ROS) production [29], and *Babesia* suppresses adaptive immunity against *Borrelia*, worsening Lyme disease severity [30]. Together, these immune evasion strategies create a feedback loop of suppression, chronic infection, and increased zoonotic risk.

The proactive monitoring of co-infections in regions with vector overlap is vital. The surveillance of multi-pathogen exposure in dogs and wildlife can provide an early warning of zoonotic threats, aiding veterinarians and public health officials in mitigating risks. Dogs are sentinel hosts in zoonotic disease ecology [31], moving between environments shared with humans and wildlife. Co-infections in dogs highlight potential spillover risks to humans [19,32], especially in regions where expanding tick and sand fly populations overlap.

Managing co-infections involving *Leishmania*, *Borrelia*, and *Babesia* necessitates an integrated, multi-vector approach targeting both sand fly and tick populations. Effective measures, such as vector control, habitat management, and innovative immune-modulating therapies that address the compounded effects of co-infections, are crucial to mitigating disease severity in dogs and reducing zoonotic transmission risks. The study by Pessôa-Pereira et al. [4] highlights the critical implications of co-infections among vector-borne pathogens that share vectors and hosts, emphasizing how these interactions accelerate disease progression and complicate treatment. In an era of shifting ecological boundaries, understanding these pathogen interactions within hosts like dogs is vital for anticipating and mitigating zoonotic risks. By integrating vector control strategies, expanding pathogen surveillance in dogs and wildlife, and advancing targeted treatment approaches for co-infected hosts, we can better manage the complex dynamics of vector-borne diseases. This holistic approach is essential to protecting the health of both animal populations and the human communities with whom they share their environments.
